# Optimization of the Production Process and Product Quality of Titanate Nanotube–Drug Composites

**DOI:** 10.3390/nano9101406

**Published:** 2019-10-02

**Authors:** Yasmin Ranjous, Géza Regdon, Klára Pintye-Hódi, Tamás Varga, Imre Szenti, Zoltán Kónya, Tamás Sovány

**Affiliations:** 1 Institute of Pharmaceutical Technology and Regulatory Affairs, University of Szeged, Eötvös u. 6., H-6720 Szeged, Hungary; yasmin.ranjous@pharm.u-szeged.hu (Y.R.); klara.hodi@pharm.u-szeged.hu (K.P.-H.); 2 Department of Applied and Environmental Chemistry, University of Szeged, Rerrich Béla tér 1., H-6720 Szeged, Hungary; tamas.varga@chem.u-szeged.hu (T.V.); imre.szenti@chem.u-szeged.hu (I.S.); konya@chem.u-szeged.hu (Z.K.); 3 Reaction Kinetics and Surface Chemistry Research Group, Hungarian Academy of Sciences-University of Szeged, Rerrich Béla tér 1, H-6720 Szeged, Hungary

**Keywords:** atenolol, hydrochlorothiazide, titanate nanotubes, composite formation, solvent selection

## Abstract

Recently, there has been an increasing interest in the application of nanotubular structures for drug delivery. There are several promising results with carbon nanotubes; however, in light of some toxicity issues, the search for alternative materials has come into focus. The objective of the present study was to investigate the influence of the applied solvent on the composite formation of titanate nanotubes (TNTs) with various drugs in order to improve their pharmacokinetics, such as solubility, stability, and bioavailability. Composites were formed by the dissolution of atenolol (ATN) and hydrochlorothiazide (HCT) in ethanol, methanol, 0.01 M hydrochloric acid or in ethanol, 1M sodium hydroxide, dimethylformamide (DMF), dimethyl sulfoxide (DMSO), respectively, and then they were mixed with a suspension of TNTs under sonication for 30 min and vacuum-dried for 24 h. The structural properties of composites were characterized by SEM, TEM, FT-IR, differential scanning calorimetry (DSC), thermogravimetric (TG) analysis, and optical contact angle (OCA) measurements. Drug release was determined from the fast disintegrating tablets using a dissolution tester coupled with a UV–Vis spectrometer. The results revealed that not only the good solubility of the drug in the applied solvent, but also the high volatility of the solvent, is necessary for an optimal composite-formation process.

## 1. Introduction

Oral administration is the main route of drug administration for a systemic effect [1]. Orally administered drugs should be released from the dosage form and dissolve before absorption. Thus, numerous attempts such as complexation, particle size reduction, solid state alternation, the application of soft gel technology, solid dispersions, using cosolvents or forming emulsions, microemulsions, micelles, polymeric micelles, liposomes, pharmaceutical salts, and pro-drugs have been made to increase the dissolution rate of the drugs in order to improve their bioavailability [2]. Particle size reduction is an easy and suitable approach to increase the dissolution rate and thus the absorption due to the increment in specific surface area [3]; however, the stabilization of particle size may be a critical issue in this approach.

Solid state dispersion is a particularly promising technique to enhance the solubility, dissolution rate, and bioavailability of poorly water-soluble drugs and to resolve the stability problems of micronized/nanonized drugs, which are dispersed in an inert solid carrier or matrix either as fine particles or molecularly [4]. This technique has numerous advantages from many aspects, such as improved stability due to the probable interactions between the drug and carrier functional groups [5], the increment of glass transition temperature of the solid dispersion matrix [6] or the displacement of crystalline structure by an amorphous form [7,8], resulting in local solubility and wettability improvement of poorly soluble drugs [9], and suppression of drug precipitation from the supersaturated solution to achieve higher solubility and dissolution rate for the metastable drug polymorphs connected to the carrier [10]. Solid dispersions may be divided into multiple classes including solid solutions, drug–carrier complexes, glassy solutions or suspensions, simple eutectic mixtures, and amorphous drug precipitates in a crystalline carrier [11]. Solid state dispersions can be prepared with various methods including the fusion process, solvent method, fusion-solvent method, spray drying, lyophilization, hot-melt extrusion, the electrospinning method, supercritical fluid technology, and spraying on beads using a fluidized-bed coating system [2]. In the solvent method, the drug and the carrier are dissolved in a suitable solvent, which will later be evaporated at an elevated temperature or under vacuum. Then, supersaturation and simultaneous precipitation of the components happens, resulting in a solid residue. Afterwards, organic and/or toxic solvents should be completely removed under vacuum. For this purpose, many sensitive techniques can be used to detect the trace amounts of solvents, such as differential scanning calorimetry (DSC), thermogravimetric (TG) analysis, or differential thermal analysis (DTA) [2]. The upsides of this method are the ability to control drug particle size by monitoring the temperature and the solvent evaporation rate [3], the capability of evaporating solvents at a lower temperature, and reduced pressure for thermolabile drugs or for frozen systems [2]. The downsides of this method are the difficulty of choosing the appropriate solvent for both the drug and the carrier, since most of the carriers are hydrophilic while the drugs are hydrophobic [12], the necessity of complete solvent removal, especially if the solvents can plasticize the carrier [13], and the large volume of solvent required to dissolve both the drug and the carrier, which is not economical in some cases [2].

Conventional drugs have many limitations, such as restricted drug solubility, undesirable pharmacodynamics, side effects, short circulating time, and lack of selectivity [14,15,16,17]. Of the drugs currently on the market, 90% are hydrophobic and poorly soluble or insoluble in water, which restricts systemic delivery [18]. However, nanocarriers may improve solubility, absorption, permeation, and retention in the target tissues, as well as the bioavailability, circulation time, and stability of drug molecules [19]. Furthermore, they may protect various drug molecules from premature degradation in the body and show higher uptake efficiency in the target cells compared to normal cells [20]. Nanotubes not only have an exemplary inner diameter of 5–6 nm, which makes them able to contain therapeutic drugs and large biological molecules, but also a large surface area and distinct outside geometry, which enable them to be modified and multi-functionalized [21].

Titanate nanotubes (TNTs) have particularly appealing characteristics, such as hydrophilicity, biocompatibility, high surface area, stable tubular structures [22], controllable dimensions, tunable geometries, surface chemistry, and the ability to modulate drug release kinetics [23]. In addition, layered titanate nanostructures have been used in several industrial applications, such as pharmaceutics, energy storage, photocatalysis, electronics, paints, and coatings. Moreover, 1D titanate nanomaterials are receiving more scientific interest, evidenced by the fact that about one new paper is published daily, according to an ISI Web of Science topic search [23]. Furthermore, TNTs can be used as drug carriers since they can load a higher amount of drug compared to carbone nanotubes (CNTs) [24].

In a previous work, 70% ethanol solution was used as a solvent to prepare composites with various drugs [25]. The selected solvent was able to improve the poor aqueous solubility of diclofenac sodium, atenolol (ATN), and hydrochlorothiazide (HCT) [26,27,28] or improve the crystallization of the highly water-soluble diltiazem HCl, while providing uniform process conditions for better comparability. Nevertheless, the result revealed that the composite formation was suboptimal for ATN and HCT. According to our hypothesis, the suboptimal solubility of the drug in the solvent, the featured crystal growth due to slow evaporation, and the intensive drug–solvent interactions may be possible explanations. The present study aims to optimize the composite formation of TNTs with atenolol (ATN) and hydrochlorothiazide (HCT), thus improving the solubility and bioavailability of the active pharmaceutical ingredients (APIs). These drugs have poor bioavailability due to different reasons. ATN belongs to the 3rd class of the Biopharmaceutical Classification System (BCS), so it has good solubility but poor permeability, which results in a lower than 50% absorption rate from the GI tract, especially if it is taken with food. HCT belongs to BCS class IV, so poor bioavailability is due to poor solubility and permeability. The successful binding of these drugs to an appropriate nanocarrier in nanocrystalline or especially in amorphous form may considerably increase their bioavailability. Therefore, the selection of the optimal solvents and process conditions is essential for such drugs.

## 2. Materials and Methods

The titanate nanotubes (TNTs), TNT-ATN (TiATN), and TNT-HCT (TiHCT) composites were prepared at the University of Szeged’s Department of Applied and Environmental Chemistry following the general composite formation method described by Sipos et al. [25]. However, since composite formation was not completely successful in the previous study, the 70% ethanol solution was replaced with methanol (0.0168% water content) and 0.01 M aqueous solution of HCl (HCl 0.01 M) or with 1 M aqueous solution of sodium hydroxide (NaOH 1M), DMF (0.012% water content), and DMSO (0.027% water content) for the synthesis of TiATN and TiHCT, respectively. The water content of the solvents was determined with Karl–Fisher titration. The solvents were purchased from Molar Chemicals Ltd., Budapest, Hungary. In addition, since TNTs exhibit instability below pH 2, TNTs treated with HCl 0.01 M were prepared as a reference to detect if this solvent may cause any change in the properties of titanate nanotubes.

ATN and HCT were kindly supplied by TEVA Pharmaceuticals PLC, Debrecen, Hungary and Gedeon Richter PLC, Budapest, Hungary, respectively. The excipients used for tablets were Avicel PH 112 (FMC Biopolymer Inc., Philadelphia, PA, USA), Tablettose 70 (Meggle Pharma GmbH, Wasserburg am Inn, Germany), talc, and magnesium stearate (both from Molar Chemicals Ltd., Budapest, Hungary).

Hydrothermally synthetized TNTs were prepared by adding 120 g of NaOH in 300 mL of distilled water on a magnetic stirrer for a few minutes and then adding 75 g of TiO_2_ for 15 min. After that, the mixture was put in the autoclave at 185 °C for 24 h then cooled at room temperature for 2 h, followed by cooling with cold water. TNTs were washed with distilled water under vacuum and by using filter No:4.

TNTs with HCl 0.01 M were prepared by adding 50 g of TNTs in 300 mL of HCl 0.01 M in an ultrasonic bath until a homogenous suspension was obtained. After that, 200 mL of HCl 0.01 M was added to the previous suspension on a magnetic stirrer and the mixture was dried in a dry oven for 24 h to remove the solvent.

A 1:1 ratio of TiATN–methanol and TiATN–HCl composites were prepared by adding 50 g of TNTs in 300 mL of methanol in an ultrasonic bath until a homogenous suspension was obtained and 50 g of atenolol in 200 mL of methanol on a magnetic stirrer. After that, the two mixtures were added to each other on the magnetic stirrer, and the final mixture was put in a vacuum distillation device until complete removal of the solvent.

To prepare a 1:1 ratio of TiHCT–NaOH composite, 50 g of TNTs were put in 1000 mL of NaOH 1 M in an ultrasonic bath to get a homogenous suspension, and 50 g of HCT was dissolved in 500 mL of NaOH 1 M on a magnetic stirrer until complete dissolution. Furthermore, the two prepared mixtures were added to each other on a magnetic stirrer until reaching homogeneity. After that, 130 mL of HCl 37% was added to neutralize the final mixture, which was washed with distilled water in a vacuum dryer until pH = 9 to eliminate the solvent. Finally, the obtained powder was dried in a dry oven for 24 h to get the required composites TiATN and TiHCT.

A 1:1 ratio of TiHCT–DMF and TiHCT–DMSO composites were prepared by adding 50 g of TNTs in 1000 mL of DMF in an ultrasonic bath and 50 g of HCT with 1000 mL of DMF on a magnetic stirrer. Then, the two prepared mixtures were added to each other on a magnetic stirrer until a homogenous mixture was obtained, which was put in a vacuum distillation device to remove the solvent.

The morphology and size of the TNTs and composites were investigated by scanning electron microscope (SEM) (Hitachi 4700, Hitachi Ltd., Tokyo, Japan) and transmission electron microscope (TEM) (FEI Tecnai G2 20 X-TWIN, Hillsboro, OR, USA). The APIs, TNTs, and the composites were coated with a thin conductive gold layer by a sputter coating unit (Polaron E5100, VG Microtech, London, UK) for the SEM measurements. The images were taken at an accelerating voltage of 10.0 kV, the used air pressure was 1.3–13 mPa during the analyses. TEM images were taken at 100 kV of electron energy, and those images served to analyze the particle size of TNTs by using Image J 1.47 t (National Institute of Health, Bethesda, MD, USA) software.

To detect the interactions between the APIs and the TNTs, a Thermo Nicolet Avatar 330 FT-IR spectrometer (Thermo Fisher Scientific Ltd., Waltham, MA, USA) was used. Measurements were performed with a Transmission E.S.P. accessory by using 256 scans at a resolution of 4 nm and applying H_2_O and CO_2_ corrections. Results were evaluated with Spectragryph 1.2.8 software (Friedrich Menges, Obersdorf, Germany). For better comparability of the original spectra of ATN and HCT with the TiATN and TiHCT composites, respectively, the signal of TNTs was subtracted from the composite spectra and the spectra were normalized to the highest peak which belongs to C=O stretching.

The surface free energy of the prepared samples was determined with a DataPhysics OCA20 (DataPhysics Instruments GmbH, Filderstadt, Germany) optical contact angle tester by using the sessile drop method. Polar and apolar test liquid (water and diiodomethane) were used and dropped onto the surface of 13-mm-diameter tablets prepared with a Specac hydraulic press (Specac Ltd., Orpington, UK) at a pressure of 3 tons. Disperse (γ_s_^D^) and polar (γ_s_^P^) components of the total surface free energy (γ_s_) of the solid were calculated according to Wu Equations (1) and (2).

(1 − cosΘ_1_)γ_1_ = 4(((γ_1_^D^γ_s_^D^)/(γ_1_^D^ + γ_s_^D^)) + ((γ_1_^P^γ_s_^P^)/(γ_1_^P^ + γ_s_^P^))), (1)

(1 − cosΘ_2_)γ_2_ = 4(((γ_2_^D^γ_s_^D^)/(γ_2_^D^ + γ_s_^D^)) + ((γ_2_^P^γ_s_^P^)/(γ_2_^P^ + γ_s_^P^))), (2)

where γ_1_ is the surface tension of the first and γ_2_ is the surface tension of the second liquid.

Polarity was calculated according to the following Equation (3):

Polarity = Уs^P^/Уs × 100. (3)

The thermal behavior of TNTs, APIs, and composites was determined by thermogravimetric analysis (TGA) and differential scanning calorimetry (DSC) analysis. TGA and DSC tests were performed by a Mettler Toledo TGA/DSC1 simultaneous analyzer (Mettler-Toledo Ltd., Budapest, Hungary) in which the samples were heated steadily from 25 to 500 °C with a heating rate of 10 K/min, using nitrogen as purge gas. The mass of the samples was 10 ± 1 mg in a closed aluminum pan (100 µL). The curves were evaluated with STARe Software (Mettler-Toledo Ltd, Budapest Hungary). To compare the curves of the API, TNTs, and the composite, the results were normalized to sample weight and to the temperature of the reference pan.

Tablets containing APIs or API–TNT composites (Table 1) were formulated to study drug release. The powders were mixed with a Turbula mixer (Willy A. Bachofen Maschinenfabrik AG, Muttenz, Switzerland) for 8 min without magnesium stearate and for an additional 2 min with it. Tablets (300 mg) containing 50 mg of API were prepared with a Korsch EK0 eccentric tablet press (E. Korsch Maschinenfabrik GmbH, Berlin, Germany) instrumented with strain gauges and a displacement transducer using 10-mm-diameter flat punches and a 5 kN compression force for all compositions.

Drug release was determined with an Erweka DT700 (Erweka GmbH, Heusenstamm, Germany) dissolution tester using the USP II method. Dissolution was applied at 37 °C using pH 1.2 enzyme-free artificial gastric juice as dissolution media. Samples of 5 mL were taken after 5 min, 10 min, 15 min, 30 min, 60 min, 90 min, and 120 min. The concentration of the released drug was measured with a ThermoScientific GENESYS 10S UV–Vis spectrophotometer (Thermo Fisher Scientific Ltd., Waltham, MA, USA), and the results were evaluated with Sigmaplot v12 (Systat Sofware Inc., San Jose, CA, USA) software.

## 3. Results

### 3.1. Properties of the TNTs

Hydrothermal synthesis produces TNTs with asymmetric, open-ended, and particular spiral cross-sectioned tubular structure [25]. The dimensions and surface characteristics of TNTs are sensitive to the synthesis conditions. Since the present work compares the properties of freshly synthetized TNTs and TNT–API composites with those from the previous work [25], the appropriate reproduction of the properties of the starting TNTs was essential.

**Figure 1:**
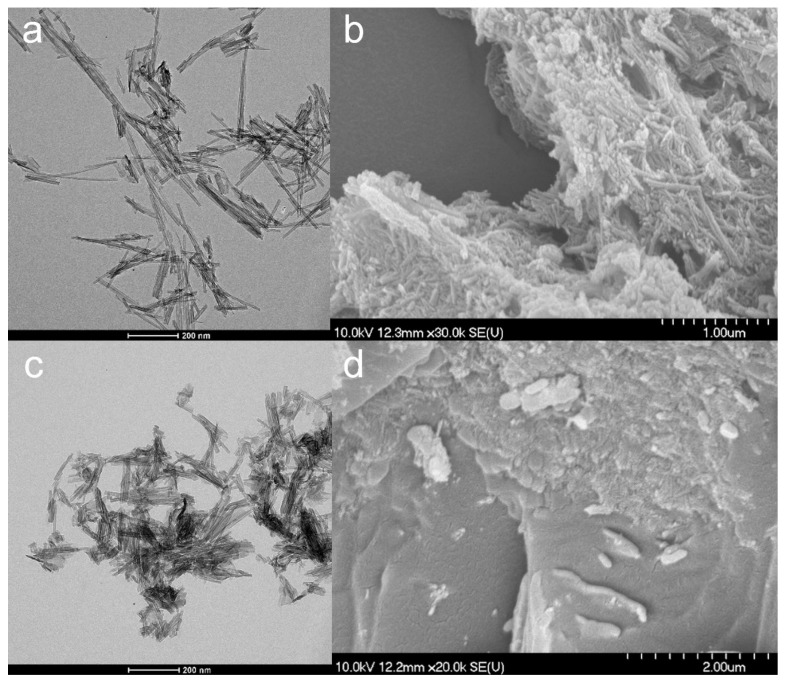
TEM (**a**,**c**) and SEM (**b**,**d**) micrographs of TNTs (**a**,**b**) and TNT–HCl 0.01 M (**c**,**d**).

According to TEM images (Figure 1a), TNTs were effectively prepared without nanowire formation. The obtained TNTs had an average length of 116.22 nm (SD ± 49.49 nm) and an average diameter of 10.99 nm (SD ± 10.15 nm), which considerably approaches the previously described results of Sipos et al. [25]. The SEM images (Figure 1b) also exhibited the characteristic aggregates of almost distinct and randomly oriented TNTs. Furthermore, the results of the contact angle measurements showed no significant difference in the surface characteristics of the new and the previous batch of TNTs (Table 2).

**Figure 2:**
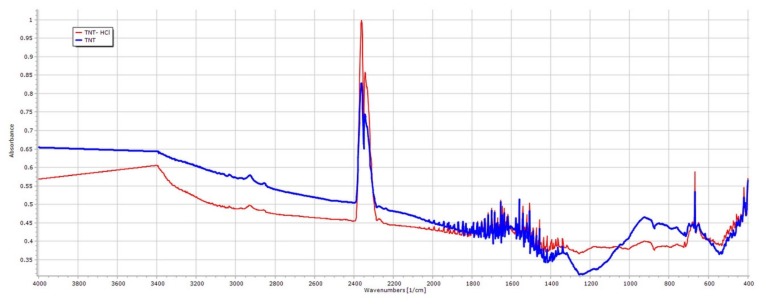
FT-IR spectra of TNTs and TNT–HCl 0.01 M.

TNT–HCl samples were also prepared as a reference to investigate the effect of diluted 0.01 M HCl on the properties of TNTs (Figure 1c,d) since strong acidic media may induce the decomposition of the nanotubular structure. The dimensions of TNT–HCl were slightly smaller than TNTs, with an average length of 83.92 nm (SD ± 42.48 nm) and an average diameter of 8.78 nm (SD ± 1.76 nm). However, neither the surface characteristics (Table 2) nor the FT-IR spectrum (Figure 2) showed a significant difference from the results of the native TNTs. Thus, no considerable difference was expected in the behavior of TNT and TNT–HCl from the aspect of composite formation ability.

### 3.2. Effect of Various Solvents on Composite Formation with ATN

**Figure 3:**
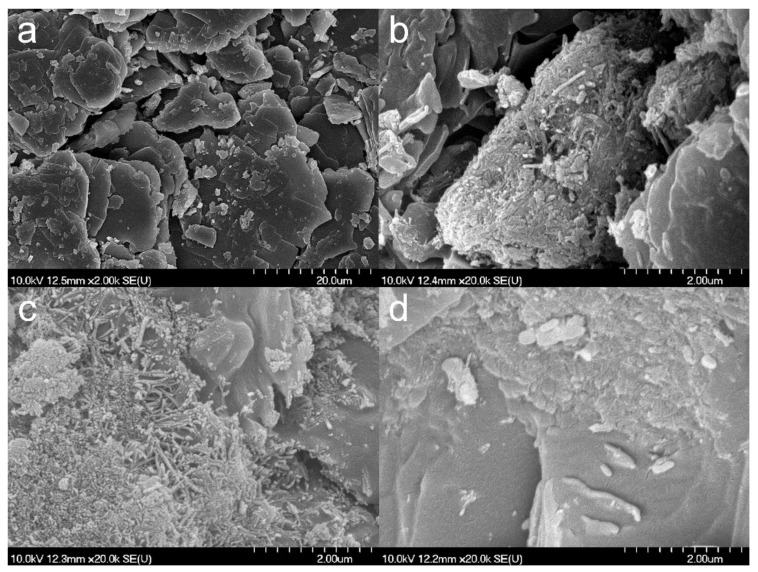
SEM micrographs of atenolol (**a**), TiATN–ethanol (**b**), TiATN–methanol (**c**), and TiATN–HCl 0.01 M (**d**).

SEM images (Figure 3) show that the composite formation was insufficient for TiATN–ethanol (Figure 3b) since the smooth-surfaced particles of crystalline ATN (Figure 3a) are clearly visible beside the aggregates of TNTs. In contrast, a strong surface coverage of ATN particles with TNTs may be observed in the micrographs of the TiATN–methanol sample (Figure 3c), indicating a stronger interaction but still insufficient composite formation between the drug and the carrier. A rough surface and highly ordered aggregations can be observed in TiATN–HCl (Figure 3d), which may indicate a more adequate composite formation and the accumulation of ATN nanocrystals on the surface of the composite without the existence of individual ATN crystals.

Interestingly, the γ_s_ (Table 1) for TiATN–ethanol and TiATN–methanol were almost identical to ATN, which may indicate not pure TNTs but TNT–ATN composites aggregated to the surface of bigger ATN particles. However, the γ_s_ and polarity values for TiATN–HCl were distinctly different from the pure ATN and TNTs, which may reflect not only a kind of interaction between NH_3_^+^ from ATN and the hydrophilic sites in TNTs enriching the hydrophobic regions in TNTs, but also a different particle-forming mechanism than in the case of other solvents, which leads to a different expected behavior during processing and use.

**Figure 4:**
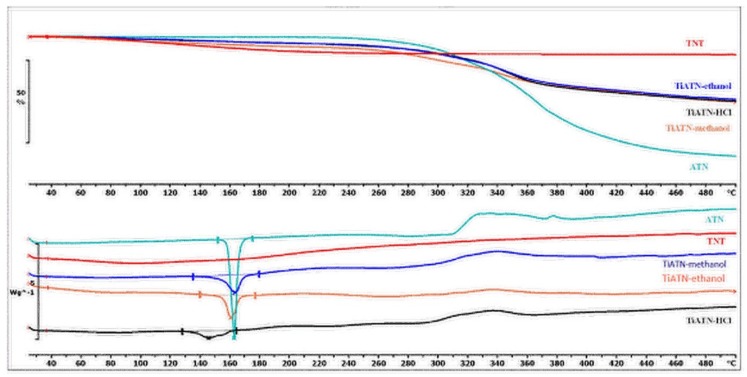
Differential scanning calorimetry (DSC) and thermogravimetric (TG) curves of TNT, ATN, TiATN–methanol, TiATN–ethanol, and TiATN–HCl.

**Figure 5:**
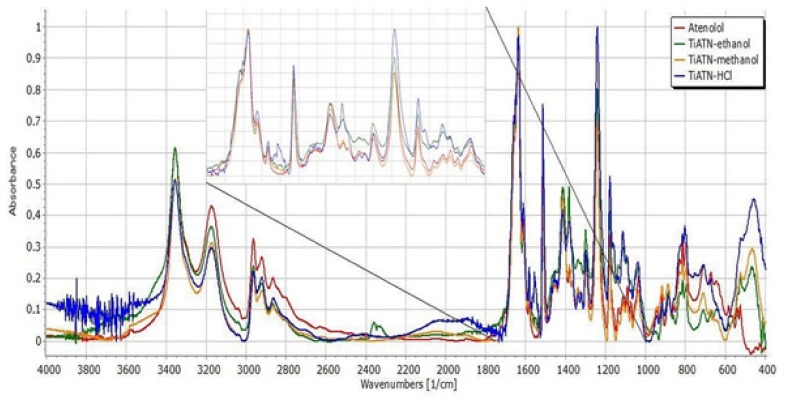
FT-IR spectra of atenolol, TiATN–ethanol, TiATN–methanol, and TiATN–HCl 0.01 M.

Nevertheless, the consequences drawn from the morphological investigations were only partially supported by the DSC/TG and FT-IR measurements. It is visible in Figure 4 that the DSC curve of ATN contains an endothermic and a broad exothermic peak. The sharp endothermic peak at 155.21 °C represents the fusion of the compound, and the exothermic peak describes its decomposition, which is supported by the TG curve of ATN. Similarly, TiATN–ethanol and TiATN–methanol composites have an endothermic peak at 161 °C and 159.84 °C, respectively. A decrease in the enthalpy of fusion was noticed from −154.795 Jg^−1^ in ATN to −74.97 Jg^−1^ and −50.71 Jg^−1^ in TiATN–ethanol and TiATN–methanol, respectively, which suggests that poor composite formation was achieved by using ethanol or methanol as solvent. Only a minor size reduction may be concluded in the case of TiATN–methanol. In contrast, the shift of the fusion temperature was higher in the TiATN–HCl composite, and the enthalpy of fusion significantly decreased to −40.55 Jg^−1^, which may be explained by the stronger interactions and by the considerable particle size reduction of ATN [29], which was in accordance with the SEM images where less crystallization was observed. These results were also supported by the FT-IR spectra, which displayed no substantial differences between the spectra of ATN, TiATN–ethanol, and TiATN–methanol (Figure 5), whereas the appearance of a new peak at 1560 cm^−1^ indicating the protonation of the carbonamide group and wide low intensity peaks between 1900–2100 cm^−1^ and 2300–2500 cm^−1^ indicating the protonation of the secondary amino group were noticed in the spectrum of TiATN–HCl.

For better comparability, the spectra of ATN and its composites were normalized to the C=O stretching peak at 1637 cm^−1^ (Figure 5). The characteristic peaks of pure ATN at 2964 cm^−1^ (C–H stretching in CH_3_), 2922 cm^−1^ (C–H stretching in CH_2_), 2865.8 cm^−1^ (C–H stretching), 1614 cm^−1^ (conjugated C=C in the aromatic ring), and 885.9 cm^−1^ (C=CH_2_ vibrations) did not show considerable differences, indicating that the C–C skeleton of the molecule is not affected by the composite formation process. However, the minor left-shift of the peak at 2800 cm^−1^ indicates the involvement of the secondary amino group in the drug carrier interactions in TiATN–HCl samples. A considerable decrease in the relative intensity at 3356 cm^−1^ can be noticed in TiATN–methanol and TiATN–HCl compared to ATN and TiATN–ethanol, which refers to the participation of the secondary –OH as a hydrogen donor in TNT–ATN conjugation. Furthermore, a similar decrease of the relative intensity of the peak at 3173 cm^−1^ may be observed for TiATN–methanol and TiATN–HCl, which indicates the participation of the carbonamide group in hydrogen bonding formation. The shift of the peak from 1637 cm^−1^ to 1650 cm^−1^ and 1659 cm^−1^ in TiATN–HCl and TiATN–ethanol samples, respectively, and the appearance of a peak at 1558 cm^−1^ in TIATN–HCl also supports the participation of the amide N as H donor and the C=O as H acceptor in the conjugation process. Moreover, the appearance of a new broad peak at around 2000 cm^−1^ indicates the formation of ATN chloride salt. The shift of the peak in TiATN–ethanol from 1382 cm^−1^ to 1385 cm^−1^, belonging to the associated β-OH vibration, indicates the role of the secondary alcohol in the conjugation. Similar, but stronger shifts of the peaks belonging to the β–OH deformation vibration were also observed in TiATN–HCl, which indicates stronger association between TNTs and ATN in this sample.

**Figure 6:**
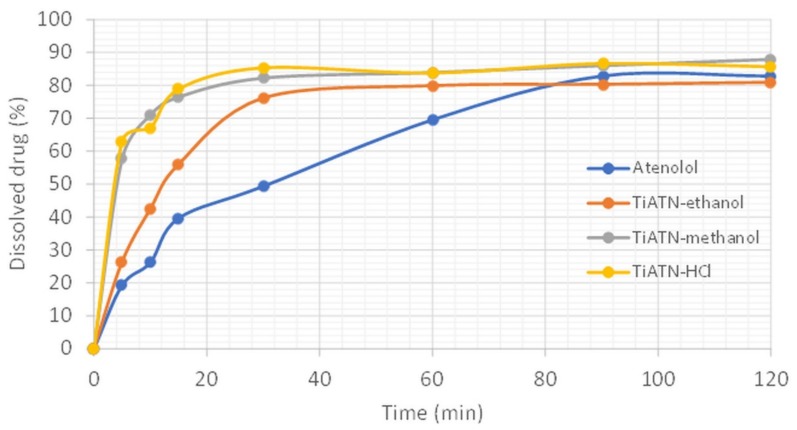
Dissolution study of TiATN composites in gastric juice (non-sink conditions).

All of these data indicate considerable solvent-dependent differences in the formation process of TiATN composites. The surface characteristics and SEM images of TiATN ethanol samples indicate that ATN nanocrystals covered Ti nanotubes and also that bigger ATN crystals may be found in the system. This may be explained by the phenomenon that the highest solubility of ATN can be observed in the 70 *w*/*w*% ethanol solution [27]. The fast evaporation of the ethanol content during solvent removal may induce the fast supersaturation of the solution, which can result in intensive nanocrystal formation on the surface of TNTs as nuclei. On the other hand, the rest of the ATN may undergo a slower crystallization process due to the slower evaporation rate of and strong H-bond-based interactions with water. Similarly, the fast evaporation of the water-free methanol featured fast nanocrystal formation on the TNTs surface and resulted in stronger composites. Nevertheless, despite the slow evaporation rate, the strongest reaction between TNTs and ATN was achieved by using HCl 0.01 M as a solvent. This may be explained by the protonation of the carboxyl amide and secondary amino groups of ATN, which results in repulsion between ATN molecules and may increase their H-bonding strength in the presence of polyfunctional carriers such as TNTs [30]. These effects also lead to featured ATN–TNT interactions, which result in an increased dissolution rate from the composites due the formation of stable nanocrystals on the carriers’ surface (Figure 6).

### 3.3. Effect of Various Solvents on the Composite Formation with HCT

**Figure 7:**
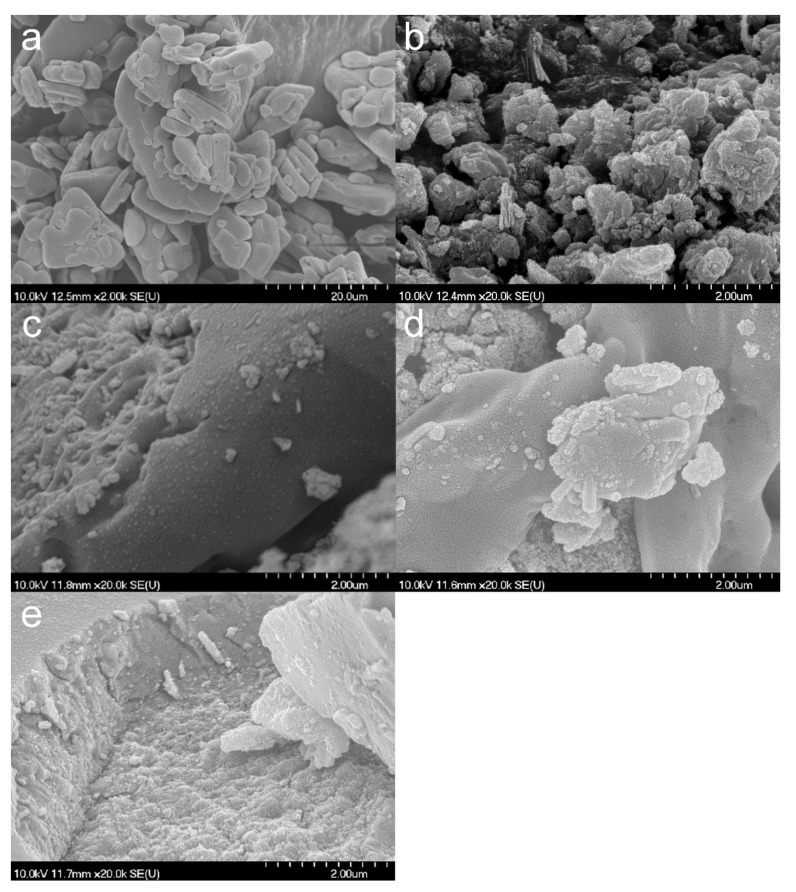
SEM micrographs of HCT (**a**), TiHCT–ethanol (**b**), TiHCT–NaOH (**c**), TiHCT–DMF (**d**), TiHCT–DMSO (**e**).

The SEM micrographs reveal a strong recrystallization of HCT (Figure 7a) from ethanol and NaOH (Figure 7b,c) with the appearance of HCT crystals covered by the composites. In contrast, in the case of TiHCT–DMF and TiHCT–DMSO (Figure 7d,e), no considerable recrystallization was observed and only strong compacts of TNTs with increased thickness are visible, which indicates an appropriate loading of the nanotubes with the API, which was also supported by the results of the OCA measurements (Table 1) and DSC analysis.

The OCA results revealed that γ_s_ and polarity values for TiHCT–ethanol and TiHCT–NaOH were higher compared to HCT due to the accumulation of TNTs on the surface of HCT crystals, whereas TiHCT–DMF and TiHCT–DMSO showed γ_s_ values similar to HCT due to the surface coverage of TNTs with HCT molecules.

**Figure 8:**
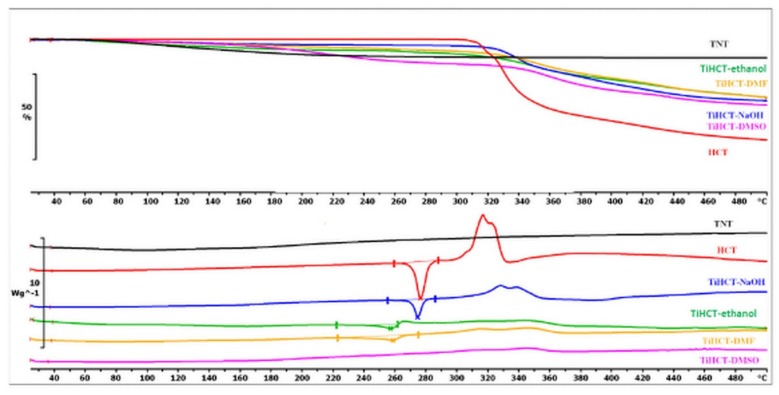
DSC and TG curves of TNT, HCT, TiHCT–ethanol, TiHCT–NaOH, TiHCT–DMF, and TiHCT–DMSO.

**Figure 9:**
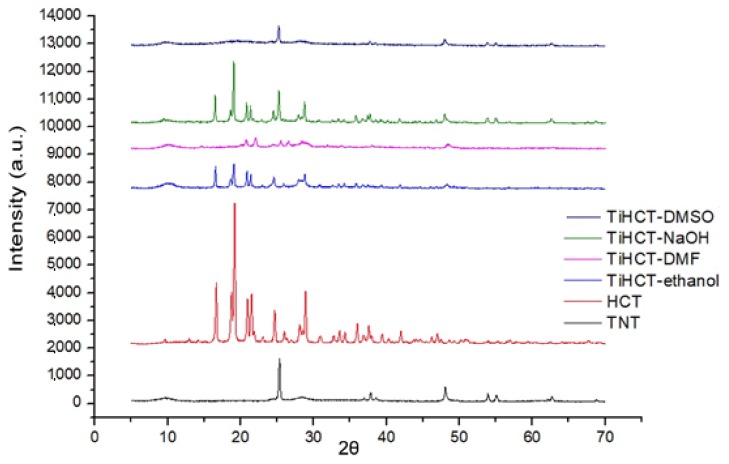
X-ray diffractograms of HCT and its composites.

The DSC curve of HCT reveals that the fusion of the API could be recognized near 270.67 °C, which was followed by a characteristic exothermic event near 320 °C, ascribed to the decomposition of the material (Figure 8). There was a slight shift in the melting peak in TiHCT–NaOH composites to 269.74 °C, which indicates poor composite formation from this solvent. This can be explained by the long evaporation time of the aqueous medium, which resulted in the domination of crystal growth and not the core formation. This effect is also strengthened by the deprotonation of the sulfonamide group, which decreases the H-bond-forming ability of HCT. A higher shift in the melting peak was noticed at 257.2 °C and 234.17 °C in TiHCT–ethanol and TiHCT–DMF, respectively; this reflects a stronger interaction when using ethanol and DMF. In the case of TiHCT–ethanol samples, the explanation may be the same as for ATN: that the fast evaporation of ethanol indicates nuclei formation, while the slow evaporation of water features the growth of HCT crystals. In contrast, despite the slower evaporation rate, an improved composite formation was expected for the water-free DMF and DMSO due to their aprotic nature, which induced the recrystallisation of HCT on the surface of TNTs as nuclei. The expectation was confirmed for DMF but interestingly, no fusion peak can be seen in the case of TiHCT–DMSO composites, which can be explained by the formation of amorphous HCT particles instead of nanocrystals. All these findings were in accordance with the results of the X-ray powder diffraction (XRPD) results (Figure 9).

It is clearly visible that the X-ray diffractogram of TiHCT–DMSO contains only the characteristic peaks of the crystalline TNTs, which clearly indicates that we were able to bond HCT to TNTs in an amorphous form, which is highly desirable to ensure an improved dissolution rate, while the other composites contained HCT in a (nano) crystalline form. Nevertheless, it is also notable that a new polymorphic form of HCT was recrystallized from DMF (the metastable Form II or DMF solvate instead of the starting stable form I) [31,32].

**Figure 10:**
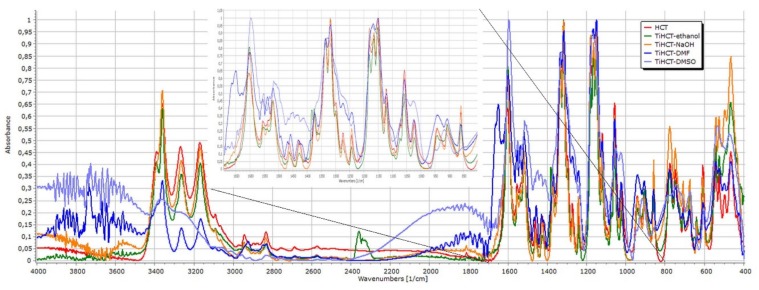
FT-IR spectra of HCT and its composites.

The spectra were normalized to the peak at 1319 cm^−1^ (Figure 10). The peaks at 3391 cm^−1^ belong to non-associated NH stretching, the right shift of this peak and that at 3269 cm^−1^ indicate the increasing strength of interactions between TNT and HCT in the order of TiHCT–ethanol, TiHCT–NaOH, TiHCT–DMF, and TiHCT–DMSO. The very strong association indicated by the merging of these peaks in the case of DMSO may also be due to the amorphous state of the drug in the composite. The slight right-shift of the N–H bend signal at 1605 cm^−1^, and the change in the intensity ratio of the peaks at 1555 cm^−1^ and 1538 cm^−1^ also indicate the participation of sulfonamide and secondary amino groups in the composite formation.

The minor modification of the C=N stretch signals between 1319–1375 cm^−1^ or the varying intensities in the 420–910 cm^−1^ region due to C–Cl stretching alteration and benzothiazidine ring skeletal vibration are also related to these interactions. Meanwhile, the left-shift and the merging of the peak triplet at 1182 cm^−1^, 1058 cm^−1^, and 1151 cm^−1^ indicate the involvement of S=O groups as hydrogen acceptors in the interactions.

**Figure 11:**
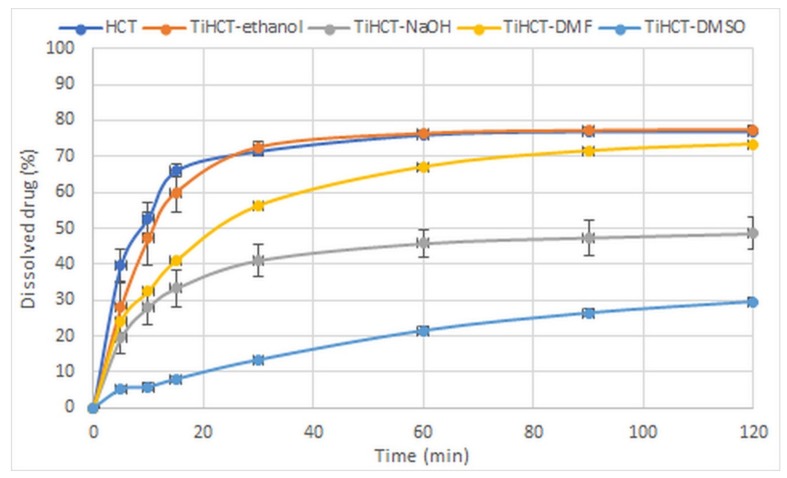
Dissolution study of TiHCT composites in gastric juice (non-sink conditions).

The strength of interactions basically determined the dissolution rate of the drug from the composites. There was no significant difference in the dissolution rates of pure HCT and TiHCT–ethanol composites where the weakest interaction was observed (Figure 11). The increasing strength of the interactions resulted in a controlled dissolution rate, which was similar to that of diclofenac–TNT composites [25] and resulted in the switch of release kinetics from first order to power law model described by Korsmeyer and Peppas. The most considerable elongation of drug release was observed in the case of TiHCT–DMSO, despite the amorphous state of the drug, which may assume an increased release rate due to the lack of a crystal lattice. It is also notable that the decrease in the release rate of TiHCT–NaOH was higher than expected, which may be due to the large size of the HCT particles due to the slow recrystallization of the drug from the solvent.

## 4. Discussion

Based on these results, it may be concluded that choosing the appropriate solvent is essential from the aspect of composite formation efficacy. The solubility of the API in the selected solvent is important as regards process performance and economy, but its volatility and protic/aprotic nature seems to be more important from the aspect of the strength and quality of composites. ATN exhibits good solubility in 70 *w*/*w*% ethanol solution, methanol, and HCl 0.01 M. The Janus-faced properties of the TiATN–ethanol sample may be explained by the fast supersaturation of the solution as a result of the fast evaporation of the ethanol content followed by the slower removal speed of water, which induced the concentration of ATN molecules and featured the formation of ATN–ATN bonds instead of ATN–TNT ones. This latter effect was not observed during the fast removal of water-free methanol, while in the case of 0.01 M HCl solution, the repulsive effect between protonated ATN molecules prohibited the formation of ATN–ATN interactions despite the slower solvent removal speed. The featured drug–carrier interactions and the stabilization of the drug in the nanocrystalline state highly improved the dissolution rate from the TiATN composites.

In contrast, the results with HCT were not so obvious. HCT was dissolved in 70 *w*/*w*% ethanol solution and NaOH 1 M as an analog of the experiments with ATN. However, the quality of the TiHCT–NaOH composites was below expectations, which may be explained by the strong interaction between the drug and the solvent, which may lead to the deprotonation of the drug molecule and may decrease the intensity of drug–carrier interactions within the composites. To eliminate this effect, a pair of aprotic solvents was applied, and DMF and DMSO were successful in forming the TiHCT composites since H-bonding was featured between HCT and TNTs due to the lack of drug–solvent interactions. Nevertheless, it is notable that despite the stabilized nanocrystalline form or HCT “recrystallizing” from DMSO in an amorphous form, the very strong drug–carrier interactions resulted in an extended release of the drug from the composites.

In conclusion, the selection of a highly volatile aprotic solvent may be the best way for the preparation of strong TNT–API composites, but the use of protic solvents could also be advantageous if it results in the protonation of the drug molecule. Nevertheless, care must be taken regarding the strength of drug–carrier interactions since they may influence the detachment of the drug and therefore exhibit considerable influence on the characteristics of the products, determining processability, release rate, and behavior in a biological environment.

## Figures and Tables

**Table 1:** Compositions of active pharmaceutical ingredient (API) and titanate nanotube (TNT)–API tablets.

Materials	API Tablets	TNT-API Tablets
API	16.7%	-
TNT–API	-	33.3%
Avicel PH 112	50.0%	39.5%
Tablettose	29.3%	23.2%
Talc	3.0%	3.0%
Mg stearate	1.0%	1.0%

**Table 2:** Surface free energy and polarity of TNTs, atenolol (ATN), hydrochlorothiazide (HCT), and their composites.

Material	Уs (mJ/m^2^)	SD	Уs^Disp^ (mJ/m^2^)	SD	Уs^Pol^ (mJ/m^2^)	SD	Polarity %
TNTs (previous)	80.72	±0.64	43.78	±0.54	36.94	±0.35	45.76
TNTs (current)	80.85	±1.18	44.55	±0.53	36.31	±1.04	44.90
TNT–HCl	78.63	±2.07	43.10	±0.27	35.53	±2.05	45.19
ATN	59.48	±3.99	36.70	±2.96	22.77	±2.68	38.20
TiATN–ethanol	60.14	±4.25	40.45	±1.48	19.68	±3.87	32.72
TiATN–methanol	58.04	±2.01	37.12	±1.19	20.92	±1.47	36.04
TiATN–HCl	68.37	±2.26	34.83	±0.05	33.54	±2.26	49.06
HCT	69.51	±2.71	43.33	±0.79	26.18	±2.59	37.60
TiHCT–ethanol	78.25	±0.86	44.65	±0.57	33.60	±0.64	42.93
TiHCT–NaOH 1M	77.54	±1.89	44.52	±0.80	33.02	±1.71	42.59
TiHCT–DMF	71.47	±2.63	42.53	±0.29	28.94	±2.63	40.49
TiHCT–DMS	73.92	±1.42	45.29	±0.08	28.63	±1.42	38.72

## References

[1] Shukla A.K., Bishnoi R.S., Dev S.K., Kumar M., Fenin V. (2017). Biopharmaceutical Classification System: Tool based prediction for drug dosage formulation. Adv. Pharm. J..

[2] Kumar P., Singh C. (2013). A study on solubility enhancement methods for poorly water soluble drugs. Am. J. Pharmacol. Sci..

[3] Habib M.J. (2000). Pharmaceutical Solid Dispersion Technology.

[4] Joshi H.N., Tejwani R.W., Davidovich M., Sahasrabudhe V.P., Jemal M., Bathala M.S., Varia S.A., Serajuddin A.T. (2004). Bioavailability enhancement of a poorly water-soluble drug by solid dispersion in polyethylene glycol–polysorbate 80 mixture. Int. J. Pharmaceut..

[5] Konno H., Taylor L.S. (2006). Influence of different polymers on the crystallization tendency of molecularly dispersed amorphous felodipine. J. Pharm. Sci..

[6] Van den Mooter G., Wuyts M., Blaton N., Busson R., Grobet P., Augustijns P., Kinget R. (2001). Physical stabilisation of amorphous ketoconazole in solid dispersions with polyvinylpyrrolidone K25. Eur. J. Pharm. Sci..

[7] Leuner C., Dressman J. (2000). Improving drug solubility for oral delivery using solid dispersions. Eur. J. Pharm. Biopharm..

[8] Yu L. (2001). Amorphous pharmaceutical solids: Preparation, characterization and stabilization. Adv. Drug Deliv. Rev..

[9] Verheyen S., Blaton N., Kinget R., van den Mooter G. (2002). Mechanism of increased dissolution of diazepam and temazepam from polyethylene glycol 6000 solid dispersions. Int. J. Pharmaceut..

[10] Martınez-Oharriz M., Martın C., Goni M., Rodrıguez-Espinosa C., Tros-Ilarduya M., Zornoza A. (1999). Influence of polyethylene glycol 4000 on the polymorphic forms of diflunisal. Eur. J. Pharm. Sci..

[11] Chiou W.L., Riegelman S. (1971). Pharmaceutical applications of solid dispersion systems. J. Pharm. Sci..

[12] Nelson E., Knoechel E., Hamlin W., Wagner J. (1962). Influence of the absorption rate of tolbutamide on the rate of decline of blood sugar levels in normal humans. J. Pharm. Sci..

[13] Lin S.L., Lachman L., Swartz C., Huebner C. (1972). Preformulation investigation I: Relation of salt forms and biological activity of an experimental antihypertensive. J. Pharm. Sci..

[14] Losic D., Simovic S. (2009). Self-ordered nanopore and nanotube platforms for drug delivery applications. Exp. Op. Drug Deliv..

[15] Aw M.S., Kurian M., Losic D. (2014). Non-eroding drug-releasing implants with ordered nanoporous and nanotubular structures: Concepts for controlling drug release. Biomaterial. Sci..

[16] Mainardes R.M., Silva L.P. (2004). Drug delivery systems: Past, present, and future. Curr. Drug Target..

[17] Fahr A., Liu X. (2007). Drug delivery strategies for poorly water-soluble drugs. Exp. Op. Drug Deliv..

[18] Wolinsky J.B., Colson Y.L., Grinstaff M.W. (2012). Local drug delivery strategies for cancer treatment: Gels, nanoparticles, polymeric films, rods, and wafers. J. Control. Rel..

[19] Pison U., Welte T., Giersig M., Groneberg D.A. (2006). Nanomedicine for respiratory diseases. Eur. J. Pharmacol..

[20] Wang S., Su R., Nie S., Sun M., Zhang J., Wu D., Moustaid-Moussa N. (2014). Application of nanotechnology in improving bioavailability and bioactivity of diet-derived phytochemicals. J. Nutr. Biochem..

[21] Kulkarni H.P. (2008). Synthesis and Applications of Titania Nanotubes: Drug Delivery and Ionomer Composites. Ph.D. Thesis.

[22] Gulati K., Ramakrishnan S., Aw M.S., Atkins G.J., Findlay D.M., Losic D. (2012). Biocompatible polymer coating of titania nanotube arrays for improved drug elution and osteoblast adhesion. Acta Biomater..

[23] Wang Q., Huang J.-Y., Li H.-Q., Zhao A.Z.-J., Wang Y., Zhang K.-Q., Sun H.-T., Lai Y.-K. (2017). Recent advances on smart TiO2 nanotube platforms for sustainable drug delivery applications. Int. J. Nanomed..

[24] Lai S., Zhang W., Liu F., Wu C., Zeng D., Sun Y., Xu Y., Fang Y., Zhou W. (2013). TiO_2_ nanotubes as animal drug delivery system and in vitro controlled release. J. Nanosci Nanotechnol..

[25] Sipos B., Pintye-Hódi K., Kónya Z., Kelemen A., Regdon G., Sovány T. (2017). Physicochemical characterisation and investigation of the bonding mechanisms of API-titanate nanotube composites as new drug carrier systems. Int. J. Pharmaceut..

[26] Saei A.A., Jabbaribar F., Fakhree M.A.A., Acree W.E., Jouyban A. (2008). Solubility of sodium diclofenac in binary water + alcohol solvent mixtures at 25 °C. J. Drug Del. Sci. Tech..

[27] Hamidi S., Jouyban A. (2015). Solubility of atenolol in ethanol + water mixtures at various temperatures. J. Serb. Chem. Soc..

[28] Wang S., Xi S., Qu Y., Wang J. (2019). Measurement and Correlation of Solubility of Hydrochlorothiazide in Monosolvents and Binary Solvent Mixtures from 283.15 to 323.15 K. J. Chem. Eng. Data.

[29] Singh M., Lara S., Tlali S. (2017). Effects of size and shape on the specific heat, melting entropy and enthalpy of nanomaterials. J. Taibah Univ. Sci..

[30] Meot-Ner M. (1984). The ionic hydrogen bond 4. Intramolecular and multiple bonds. Protonation and complexes of amides and amino acid derivatives. J. Am. Chem. Soc..

[31] Johnston A., Florence A.J., Shankland N., Kennedy A.R., Shankland K., Price S.L. (2007). Crystallization and Crystal Energy Landscape of Hydrochlorothiazide. Cryst. Growth Des..

[32] Saini A., Chadha R., Gupta A., Singh P., Bhandari S., Khullar S., Mandal S., Jain D.D. (2016). New conformational polymorph of hydrochlorothiazide with improved solubility. Pharm. Dev. Techn..

